# Plenary 3 Health Systems Performance Assessment for Policy: Uses and Abuses

**DOI:** 10.1093/eurpub/ckac128.003

**Published:** 2022-10-25

**Authors:** Organised by: European Observatory on Health Systems and Policies, European Commission, Moderators: Isabel de la Mata (European Commission), Dimitra Panteli (European Observatory on Health Systems and Policies)

## Abstract

The COVID-19 pandemic constitutes a powerful reminder of the importance of health systems strengthening in protecting and improving the health of our populations. For policy makers ‘to build back better’ systems to face future shocks, they will need to be able to determine which areas work best (in terms of providing access, quality, population health, responsiveness or efficiency) to prioritize and direct resources towards. More than ever, we need now to count with strong health systems monitoring, appraisal and assessment to draw practical policy implications.

This plenary will look at the effective application of Health Systems Performance Assessment (HSPA) to health systems’ improvement as we face key challenges in the sustainability of our health systems. Too often the results of HSPA exercises, particularly when benchmarking is involved, are not well interpreted, and understood. When translating HSPA results into policy changes, we need to address a series of questions not only about the quality and validity of the indicators but, importantly, about the causal attribution and accountability implications and about the kinds of policy interventions required to address the performance failures.

Following an introductory keynote providing practical illustrations of those issues and drawing policy lessons, the panelists will focus on three key HSPA questions particularly relevant in the current policy context. First, how to interpret and attribute overall health system performance outcomes to individual health system functions and strategies in need of reform. Second, how best to benchmark and compare performance between European countries to identify and learn from best practices. Finally, how to measure resilience as core component of systems performance and draw lessons to prepare for future shocks.

**Introductory keynote speaker:**

**Reinhard Busse**

European Observatory on Health Systems and Policies and Technical University Berlin, Germany

**Panellists:**

**Kenneth Grech**

EU Expert Group Health Systems Performance Assessment & Ministry of Health Malta, Malta

**Irini Papanicolas**

Department of Health Policy, London School of Economics and Political Science, UK

**Marina Karanikolos**

Research Fellow, European Observatory on Health Systems and Policies; London School of Economics and Political Science, UK

